# Lipid-Based Nanoformulations of [6]-Gingerol for the Chemoprevention of Benzo[a] Pyrene-Induced Lung Carcinogenesis: Preclinical Evidence

**DOI:** 10.3390/ph18040574

**Published:** 2025-04-15

**Authors:** Faris Alrumaihi, Ali Yousif Babiker, Arif Khan

**Affiliations:** 1 Department of Medical Laboratories, College of Applied Medical Sciences, Qassim University, Buraydah 51452, Saudi Arabia; f_alrumaihi@qu.edu.sa (F.A.); ababkr@qu.edu.sa (A.Y.B.); 2 Department of Basic Health Sciences, College of Applied Medical Sciences, Qassim University, Buraydah 51452, Saudi Arabia

**Keywords:** lung cancer, animal model, cancer therapy, drug delivery system, drug formulation, nanocarrier

## Abstract

**Background/Objectives:** [6]-Gingerol ([6]-G), a bioactive compound derived from Zingiber officinale (ginger), exhibits strong anticancer potential but is hindered by poor aqueous solubility and low bioavailability. This study aimed to develop and evaluate PEGylated liposomal [6]-G (6-G-Lip) to enhance its stability, bioavailability, and chemopreventive efficacy in benzo[a]pyrene (BaP)-induced lung carcinogenesis. **Methods:** 6-G-Lip was synthesized using a modified thin-film hydration technique and characterized for size, polydispersity index (PDI), zeta potential, encapsulation efficiency (EE%), and release kinetics. The chemopreventive effects were assessed in BaP-induced lung cancer in Swiss albino mice, with prophylactic 6-G-Lip administration from two weeks before BaP exposure through 21 weeks. Cancer biomarkers, antioxidant enzyme activity, reactive oxygen species (ROS) generation, induction of apoptosis, and histopathological alterations were analyzed. **Results:** 6-G-Lip exhibited a particle size of 129.7 nm, a polydispersity index (PDI) of 0.16, a zeta potential of −18.2 mV, and an encapsulation efficiency (EE%) of 91%, ensuring stability and effective drug loading. The formulation exhibited a controlled release profile, with 26.5% and 47.5% of [6]-G released in PBS and serum, respectively, at 72 h. 6-G-Lip significantly lowered cancer biomarkers, restored antioxidant defenses (SOD: 5.60 U/min/mg protein; CAT: 166.66 μm H_2_O_2_/min/mg protein), reduced lipid peroxidation (MDA: 3.3 nm/min/mg protein), and induced apoptosis (42.2%), highlighting its chemopreventive efficacy. **Conclusions:** This study is the first to prepare, characterize, and evaluate PEGylated [6]-G-Lip for the chemoprevention of lung cancer. It modulates oxidative stress, restores biochemical homeostasis, and selectively induces apoptosis. These findings support 6-G-Lip as a promising nanotherapeutic strategy for cancer prevention.

## 1. Introduction

Lung cancer remains a global health crisis characterized by high incidence rates and poor survival outcomes. According to GLOBOCAN 2020, lung cancer accounts for 11.4% of all newly diagnosed cancers worldwide, with over 2.2 million new cases and approximately 1.8 million deaths recorded in a single year. With lung cancer accounting for nearly 18% of all cancer-related fatalities, it remains the foremost cause of cancer mortality worldwide, underscoring the urgent need for more effective preventive and therapeutic strategies [1]. Despite significant advancements in diagnostic techniques and treatment modalities, the survival rates for lung cancer remain exceptionally low, particularly in cases diagnosed at advanced stages [2,3,4]. In Saudi Arabia, the rising incidence of lung cancer poses an escalating public health challenge, primarily due to the increasing prevalence of tobacco use among both men and women. Smoking remains the dominant risk factor, accounting for approximately 85% of all lung cancer cases. Epidemiological data indicate that smokers have a 20-fold higher risk of developing lung cancer compared to non-smokers, reinforcing the urgent need for effective preventive strategies [5,6,7,8,9].

Among the various carcinogenic agents associated with smoking, benzo[a]pyrene (BaP), a polycyclic aromatic hydrocarbon (PAH), is a well-established environmental carcinogen with potent tumorigenic effects. BaP is generated through the incomplete combustion of organic materials, including tobacco, coal, and fossil fuels, and is classified as a Group 1 human carcinogen by the International Agency for Research on Cancer (IARC) [10]. Once inhaled, BaP undergoes metabolic activation by cytochrome P450 enzymes, specifically CYP1A1 and CYP1B1, leading to the formation of benzo[a]pyrene diol epoxide (BPDE). This highly reactive metabolite binds covalently to DNA. This process induces genomic instability, causing mutations in critical tumor suppressor genes such as p53 and KRAS, which are frequently altered in lung cancer [11,12,13]. Additionally, BaP induces oxidative stress by generating excessive reactive oxygen species (ROS), which disrupts cellular redox balance, damages DNA, and activates multiple oncogenic pathways, including mitogen-activated protein kinase (MAPK), PI3K/Akt, and NF-κB, thereby promoting uncontrolled proliferation, resistance to apoptosis, and tumor progression [14,15]. Given its well-established role in lung carcinogenesis, BaP-induced lung cancer models serve as reliable preclinical systems for evaluating novel chemopreventive interventions [16,17].

Despite ongoing advancements in lung cancer therapeutics, standard treatment modalities—including chemotherapy, radiotherapy, and targeted therapies—are often hampered by severe toxicities, limited efficacy, and the emergence of drug resistance. Many chemotherapy regimens, while initially effective, exhibit high relapse rates, leading to disease recurrence and metastasis [18]. Additionally, the heterogeneous nature of lung cancer subtypes poses a significant challenge in achieving personalized treatment responses, further complicating patient management [19,20]. These limitations have sparked increased interest in nutraceuticals—bioactive compounds derived from natural sources—as alternative or complementary strategies for cancer prevention and treatment. Nutraceuticals have demonstrated multifaceted anticancer properties, often exerting minimal toxicity while targeting multiple oncogenic pathways, making them ideal candidates for long-term preventive applications [17,21].

Among nutraceuticals, [6]-gingerol (C_17_H_26_O_4_), the principal bioactive component of ginger (*Zingiber officinale*), has emerged as a potent anticancer agent due to its anti-inflammatory, antioxidant, and antiproliferative properties [22]. Preclinical studies indicate that [6]-gingerol effectively inhibits tumor cell proliferation, induces apoptosis, and modulates key oncogenic signaling cascades, including NF-κB, MAPK, AMPK, and AKT/mTOR, while also suppressing JAK/STAT-mediated immune evasion mechanisms [23,24,25,26]. Our previous research also demonstrated the protective effects of diallyl sulfide (DAS) in BaP-induced lung carcinogenesis and its role in modulating fatty acid synthase (FASN), a critical factor in cancer metabolism [27]. Additionally, [6]-gingerol is a potent scavenger of reactive oxygen species (ROS), thereby mitigating oxidative stress-induced DNA damage, a fundamental driver of lung cancer progression. Despite these promising preclinical findings, its clinical application remains limited due to poor aqueous solubility, rapid metabolism, and low systemic bioavailability, necessitating the development of advanced drug delivery systems to optimize its therapeutic efficacy [28].

To overcome these pharmacokinetic limitations, liposomal nanoformulations have emerged as a promising strategy for drug delivery, enabling improved solubility, prolonged circulation times, and enhanced tumor targeting. Liposomes—spherical vesicles composed of phospholipid bilayers—can encapsulate hydrophilic and hydrophobic agents, effectively protecting bioactive compounds from enzymatic degradation and premature clearance [29,30,31]. The incorporation of polyethylene glycol (PEG), known as PEGylation, further enhances liposomal stability by reducing immune recognition, increasing circulation time, and improving passive tumor accumulation via the enhanced permeability and retention (EPR) effect [31,32,33]. Our research group has successfully developed and characterized PEGylated liposomal formulations of several bioactive compounds, including thymoquinone (C_17_H_26_O_4_), diallyl sulfide (C_6_H_10_S), diallyl disulfide (C_6_H_10_S_2_), and diallyl trisulfide (C_6_H_10_S_3_), which have demonstrated superior bioavailability, prolonged stability, and enhanced antitumor activity in preclinical cancer models. These nanoformulations optimize drug delivery, reduce systemic toxicity, and improve therapeutic efficacy, making them an attractive platform for cancer prevention and treatment [34,35,36,37,38].

Leveraging recent advancements in nanotechnology, liposomal encapsulation provides a cutting-edge approach to optimizing the therapeutic efficacy of [6]-gingerol, enhancing its bioavailability, stability, and targeted delivery for improved cancer prevention. Given our expertise in developing PEGylated liposomal nanocarriers, this study aims to formulate and evaluate a PEGylated [6]-gingerol liposomal system (6-G-Lip) for its chemopreventive efficacy in a BaP-induced lung cancer model. By integrating nanotechnology-driven drug delivery with nutraceutical-based cancer prevention, this research aims to optimize therapeutic outcomes, minimize toxicity, and provide a novel and effective strategy for preventing lung cancer.

## 2. Results

### 2.1. Characterization of Liposomes

#### 2.1.1. Particle Size, Polydispersity Index (PDI), Zeta Potential, and Encapsulation Efficiency (EE)

**Figure 1:**
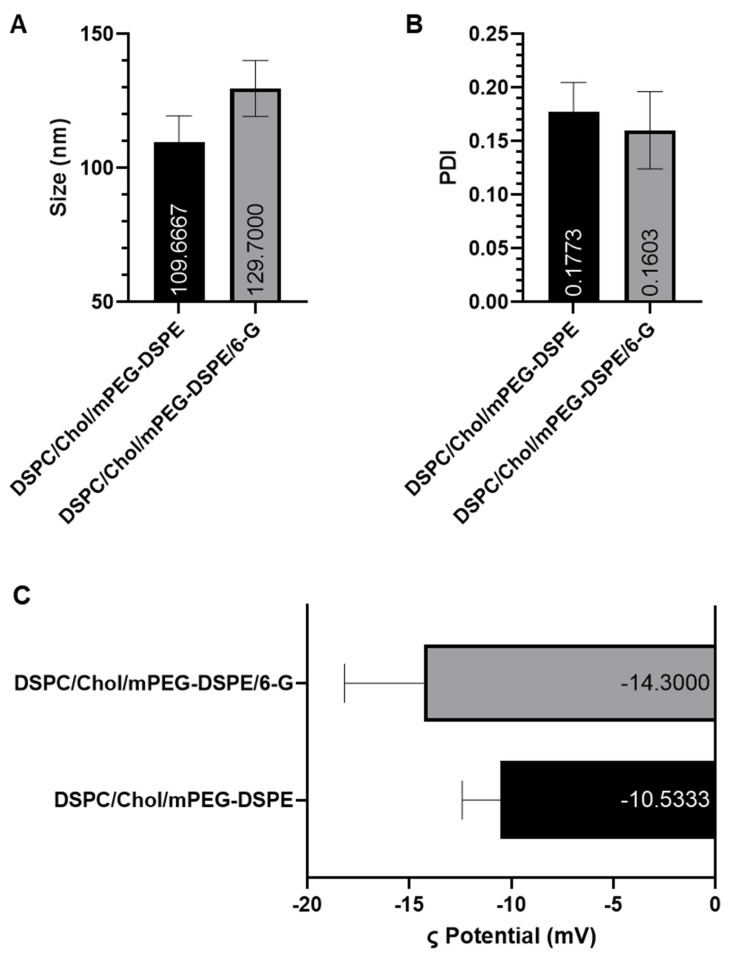

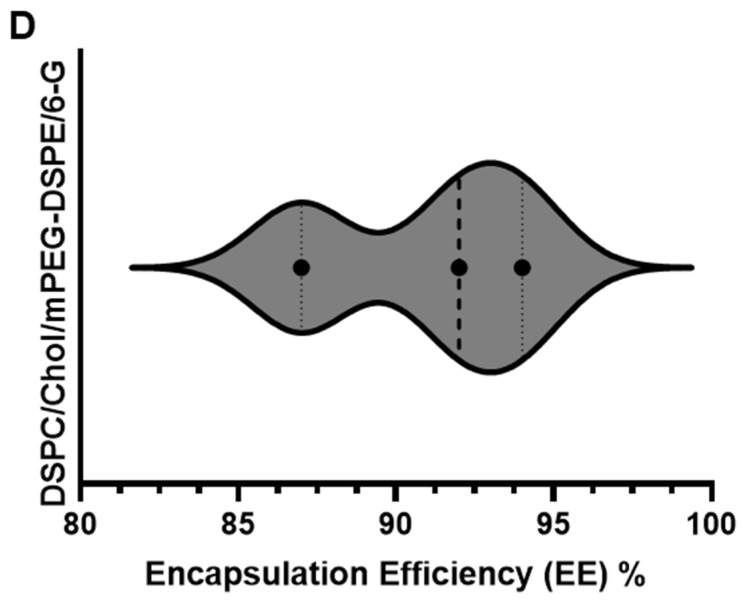
Characterization of DSPC/Chol/mPEG-DSPE and DSPC/Chol/mPEG-DSPE/6-G Liposomes. (**A**) Particle size distribution, (**B**) polydispersity index (PDI), (**C**) zeta (ζ) potential, and (**D**) encapsulation efficiency (EE%). Data are presented as mean values, with error bars representing the 95% confidence intervals (CIs) from three independent experiments.

Dynamic light scattering (DLS) analysis confirmed that PEGylated sham liposomes (DSPC/Chol-based) exhibited an average particle size of 109.7 nm (95% CI: 99.84–119.5) with a polydispersity index (PDI) of 0.18 (95% CI: 0.15–0.19), indicating a highly uniform and monodisperse formulation. The [6]-G-Lip formulation, optimized for drug delivery, displayed a slightly larger particle size of 129.7 nm (95% CI: 119.30–140.15) with a PDI of 0.16 (95% CI: 0.13–0.19), maintaining a stable nanoscale distribution (Figure 1A,B). The zeta potential values of −10.5 mV for sham liposomes and −18.2 mV for [6]-G-Lip reflect substantial electrostatic repulsion, which plays a crucial role in colloidal stability by preventing aggregation and maintaining dispersion in physiological environments (Figure 1C). The encapsulation efficiency (EE%) of [6]-G-Lip was determined to be 91% (95% CI: 84.05–95.65), demonstrating highly efficient drug entrapment within the liposomal bilayers. The high encapsulation efficiency suggests that the formulation effectively retains [6]-gingerol, supporting its suitability for therapeutic applications (Figure 1D). The slight variations observed in particle size and zeta potential between the sham and [6]-gingerol-loaded liposomes reflect the expected physicochemical influence of drug incorporation and remain well within the optimal range for colloidal stability, uniformity, and biological applicability.

#### 2.1.2. In Vitro Stability and Release Kinetics

**Figure 2:**
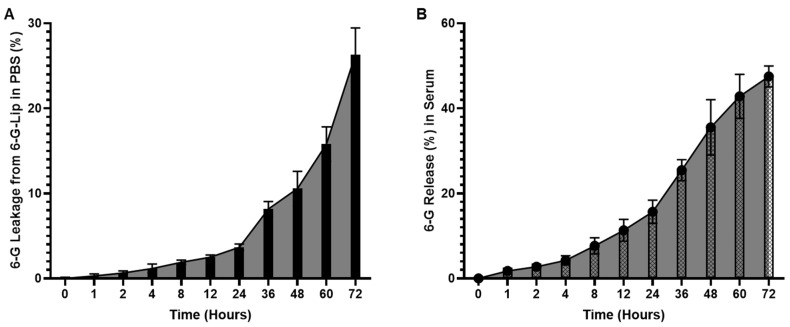
In vitro stability and release kinetics of [6]-Gingerol liposomes (6-G-Lip). (**A**) Stability of 6-G-Lip in phosphate-buffered saline (PBS) at 37 °C over 72 h, demonstrating structural integrity and minimal aggregation. (**B**) The cumulative release profile of [6]-Gingerol from PEGylated liposomes in serum under physiological conditions indicates controlled and sustained drug release. Data represent the mean ± 95% confidence intervals (CIs) of three independent experiments.

The stability of [6]-G-Lip was assessed under physiological conditions to evaluate its ability to retain the encapsulated drug. After 24 h of incubation in PBS (pH 7.4, 37 °C), less than 4% of [6]-gingerol was released, which increased to approximately 10.5% at 48 h. The cumulative drug release reached 26.5% (95% CI: 23.20–29.45) at 72 h, demonstrating a controlled and sustained release profile (Figure 2A). In serum-based release studies, the formulation exhibited a gradual and sustained release pattern, likely due to interactions between serum proteins and the PEGylated lipid bilayer. The percentage of [6]-gingerol released at 24, 48, and 72 h was 15.7% (95% CI: 12.92–18.41), 35.53% (95% CI: 29.00–42.06), and 47.5% (95% CI: 45.01–49.98), respectively (Figure 2B). These findings indicate that [6]-G-Lip maintains prolonged drug retention, which is essential for ensuring extended systemic circulation and sustained therapeutic action. The delayed release profile suggests that PEGylation effectively minimizes premature drug leakage, allowing for prolonged drug availability at the target site. This controlled release mechanism supports the potential of [6]-G-Lip in reducing dosing frequency while enhancing therapeutic efficacy.

### 2.2. Protective Effects of 6-G-Lip on BaP-Induced Body Weight Changes and Survival Rates

**Figure 3:**
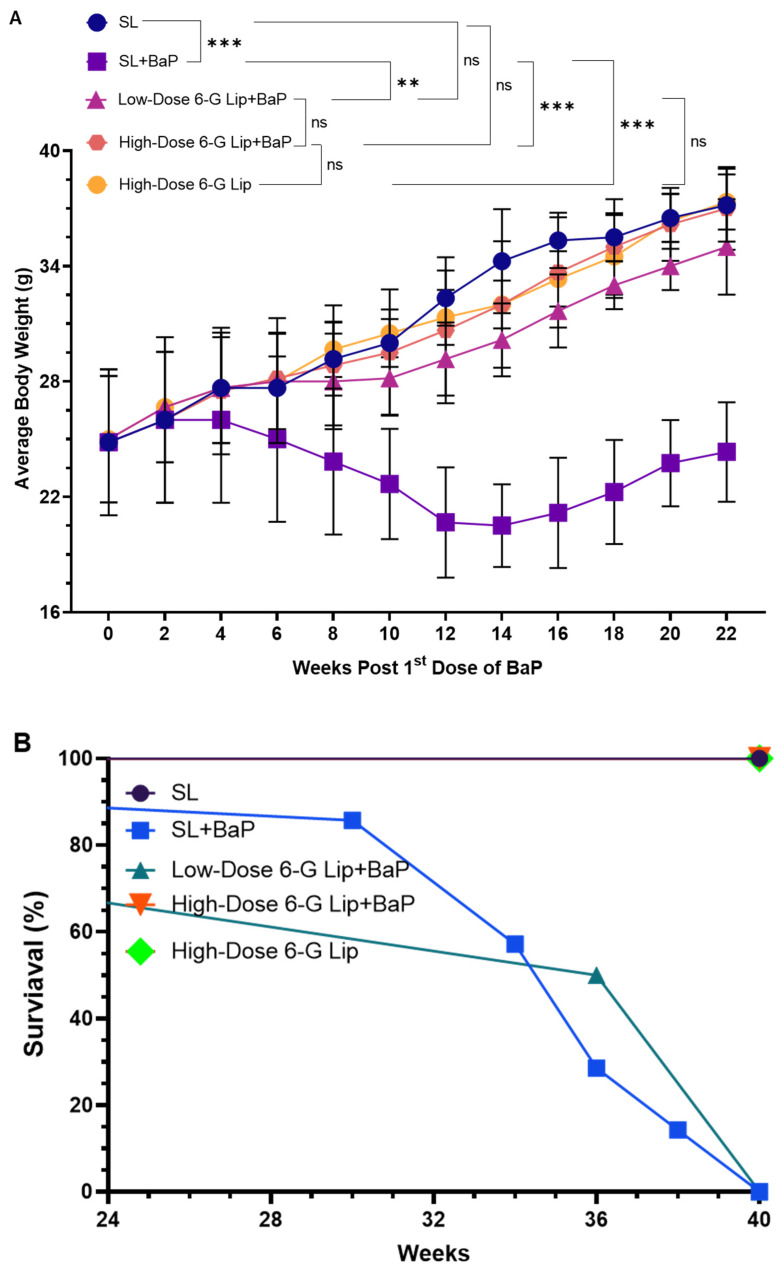
Effect of 6-G-Lip on BaP-induced changes in body weight and survival rates. (**A**) Average body weight (ABW) variations across experimental groups over the study period. Data are expressed as mean ± 95% confidence intervals (CIs), with a sample size of fifteen mice per group (n = 15). Statistical significance for ABW differences was determined using one-way ANOVA, followed by Tukey’s post hoc test. ‘ns’ indicates no statistically significant difference between groups. Asterisks denote statistical significance: ** *p* < 0.01 and *** *p* < 0.001. (**B**) Kaplan–Meier survival analysis depicting survival probabilities monitored for up to 40 weeks, with ten mice per group (n = 10). The survival differences were analyzed using the log-rank (Mantel–Cox) test.

BaP exposure significantly declined average body weight (ABW) in the SL + BaP group, which did not receive 6-G-Lip treatment. The ABW dropped from an initial 34.25 g (95% CI: 31.54–36.95) to 20.5 g (95% CI: 18.35–22.65) by week 14. Although some recovery was observed by week 22, reaching 24.4 g (95% CI: 21.75–26.90), body weights in this group remained significantly lower compared to SL (*p* < 0.01), low-dose 6-G-Lip + BaP (*p* < 0.1), high-dose 6-G-Lip + BaP (*p* < 0.01), and high-dose 6-G-Lip (*p* < 0.01). In contrast, body weights in the SL and high-dose 6-G-Lip groups remained stable at 37.25 g (95% CI: 35.26–39.06) and 37.5 g (95% CI: 35.89–38.76), respectively (Figure 3A). The gradual increase in body weight observed in the SL and chemoprevented groups is consistent with the typical physiological growth pattern of Swiss albino mice. While they are considered adults at around 8–10 weeks of age, this strain continues to gain weight steadily until 32–40 weeks, eventually stabilizing at a weight of 40–44 g under standard laboratory conditions. As all animals were housed under identical conditions, with uniform access to diet and care, the observed weight gain is attributed to normal biological development rather than overfeeding or experimental variation. This progression also reflects the absence of carcinogen-induced metabolic stress in these groups. Treatment with 6-G-Lip effectively counteracted BaP-induced weight loss in a dose-dependent manner. Notably, the high-dose 6-G-Lip + BaP group maintained a stable ABW of 37.0 g (95% CI: 34.85–39.15), closely matching the SL and high-dose 6-G-Lip groups. This suggests a strong protective effect of 6-G-Lip against BaP-induced metabolic alterations, contributing to weight reduction. Survival analysis further underscored the protective efficacy of 6-G-Lip. The Kaplan–Meier survival curve (Figure 3B) revealed that the SL + BaP group exhibited the highest mortality rate (70%), consistent with the severe impact of BaP-induced carcinogenesis. However, 6-G-Lip administration significantly improved survival outcomes in a dose-dependent manner. Mice receiving the low-dose 6-G-Lip + BaP exhibited a substantially lower mortality rate (20%), while those in the high-dose 6-G-Lip + BaP group demonstrated complete protection, achieving a 100% survival rate. These findings highlight the ability of 6-G-Lip to mitigate the detrimental effects of BaP exposure, reinforcing its potential as a promising chemopreventive intervention.

### 2.3. Modulation of Cancer Marker Enzymes in Serum

**Figure 4:**
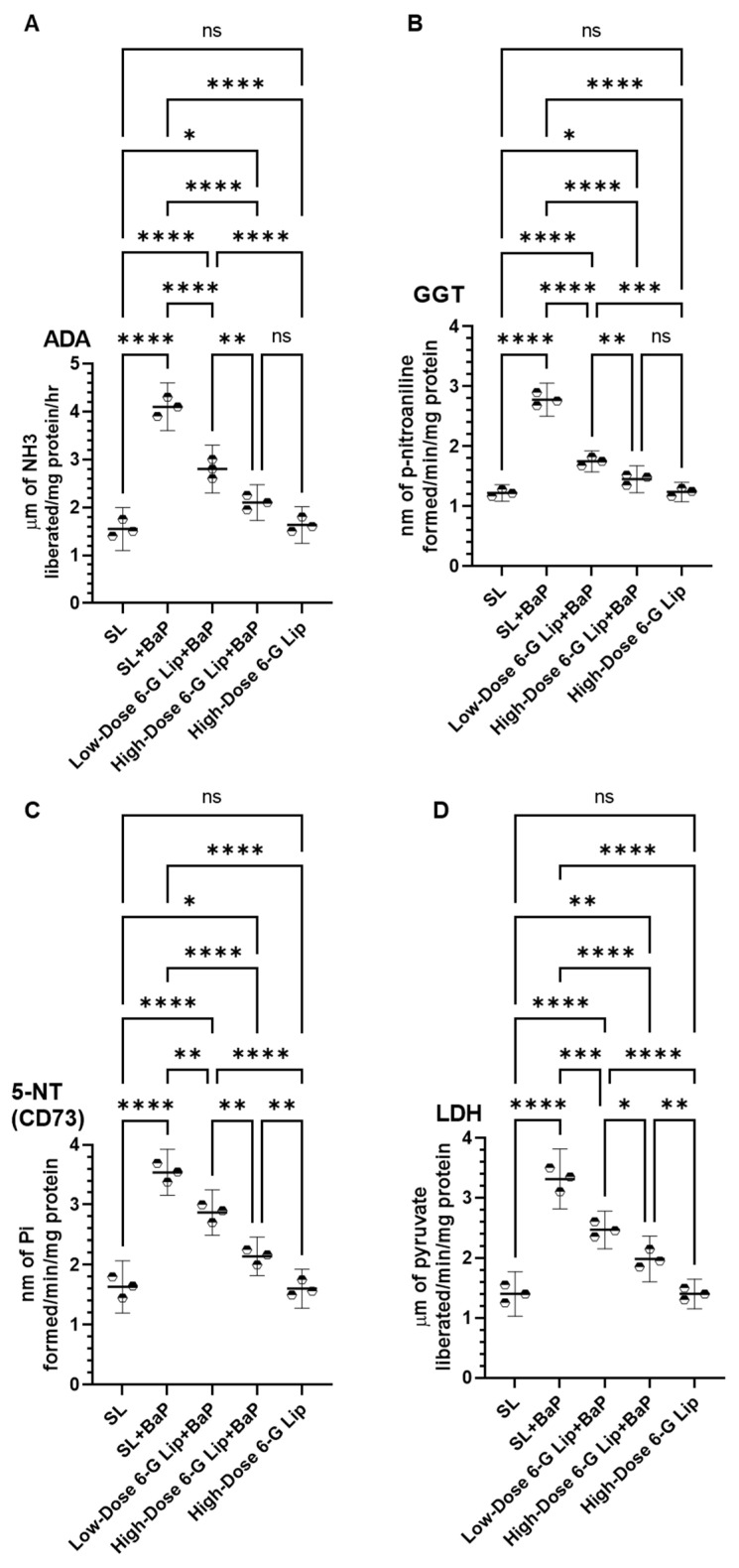
Effects of 6-G-Lip on serum cancer biomarkers in BaP-induced lung carcinogenesis. This figure illustrates the impact of 6-G-Lip on serum levels of key cancer-associated enzymes in the BaP-induced lung cancer model. (**A**) Adenosine deaminase (ADA), (**B**) Gamma-glutamyl transferase (GGT), (**C**) 5′-Nucleotidase (CD73), and (**D**) Lactate dehydrogenase (LDH) activity levels across experimental groups. BaP exposure (SL + BaP) resulted in a significant increase in these biomarkers compared to the vehicle control (SL), indicating metabolic alterations associated with tumor progression. Treatment with 6-G-Lip resulted in a dose-dependent decrease in these enzyme levels, with high-dose 6-G-Lip + BaP restoring values closer to those of the control group, suggesting its protective role in counteracting BaP-induced metabolic dysregulation. The high-dose 6-G-Lip-only group exhibited no significant differences from the SL control, confirming the formulation’s safety under physiological conditions. These findings highlight the potential of 6-G-Lip in mitigating tumor-associated metabolic disturbances and reinforce its role in chemoprevention. Data are presented as mean ± 95% confidence intervals (CIs) from three independent experiments. Statistical significance is indicated as (*) *p* < 0.05, (**) *p* < 0.01, (***) *p* < 0.001, and (****) *p* < 0.0001; ‘ns’ denotes no statistically significant differences between groups.

Exposure to BaP in the SL + BaP group resulted in a significant elevation of serum cancer marker enzymes, including adenosine deaminase (ADA), gamma-glutamyltransferase (GGT), 5′-nucleotidase (CD73), and lactate dehydrogenase (LDH), indicating heightened metabolic activity and potential carcinogenic progression. The recorded serum levels in the SL + BaP group were 4.1 μm of NH_3_ liberated/mg protein/hr (95% CI: 3.60–4.59) for ADA, 2.78 nm of p-nitroaniline formed/min/mg protein (95% CI: 2.50–3.05) for GGT, 3.54 nm of Pi formed/min/mg protein (95% CI: 3.15–3.92) for CD73, and 3.31 μm of pyruvate liberated/min/mg protein (95% CI: 2.81–3.81) for LDH. In contrast, the SL group exhibited baseline levels of 1.55 μm NH_3_ liberated/mg protein/hr (95% CI: 1.10–1.99) for ADA, 1.22 nm of p-nitroaniline formed/min/mg protein (95% CI: 1.08–1.36) for GGT, 1.62 nm of Pi formed/min/mg protein (95% CI: 1.19–2.07) for CD73, and 1.40 μm of pyruvate liberated/min/mg protein (95% CI: 1.02–1.77) for LDH (Figure 4A–D). Administration of [6]-G-Lip significantly reduced these elevated levels in a dose-dependent manner. In the low-dose 6-G-Lip + BaP group, ADA levels declined to 2.8 μm of NH_3_ liberated/mg protein/hr (95% CI: 2.30–3.29), while a further reduction to 2.1 μm of NH_3_ liberated/mg protein/hr (95% CI: 1.72–2.47) was observed in the high-dose 6-G-Lip + BaP group. Similarly, GGT levels decreased to 1.75 nm of p-nitroaniline formed/min/mg protein (95% CI: 1.57–1.92) and 1.45 nm of p-nitroaniline formed/min/mg protein (95% CI: 1.22–1.67), CD73 levels declined to 2.86 nm of Pi formed/min/mg protein (95% CI: 2.48–3.24) and 2.14 nm of Pi formed/min/mg protein (95% CI: 1.82–2.45), and LDH levels were reduced to 2.46 μm of pyruvate liberated/min/mg protein (95% CI: 2.14–2.77) and 1.98 μm of pyruvate liberated/min/mg protein (95% CI: 1.60–2.36) in the low-dose and high-dose 6-G-Lip + BaP groups, respectively. The observed reductions in these biochemical markers following [6]-G-Lip treatment support its efficacy in mitigating BaP-induced metabolic disruptions and carcinogenesis. These findings indicate that [6]-G-Lip is protective in regulating cancer-associated enzymatic alterations, reinforcing its potential as a chemopreventive intervention (Figure 4A–D).

### 2.4. Antioxidant Enzyme Activity and Lipid Peroxidation

**Figure 5:**
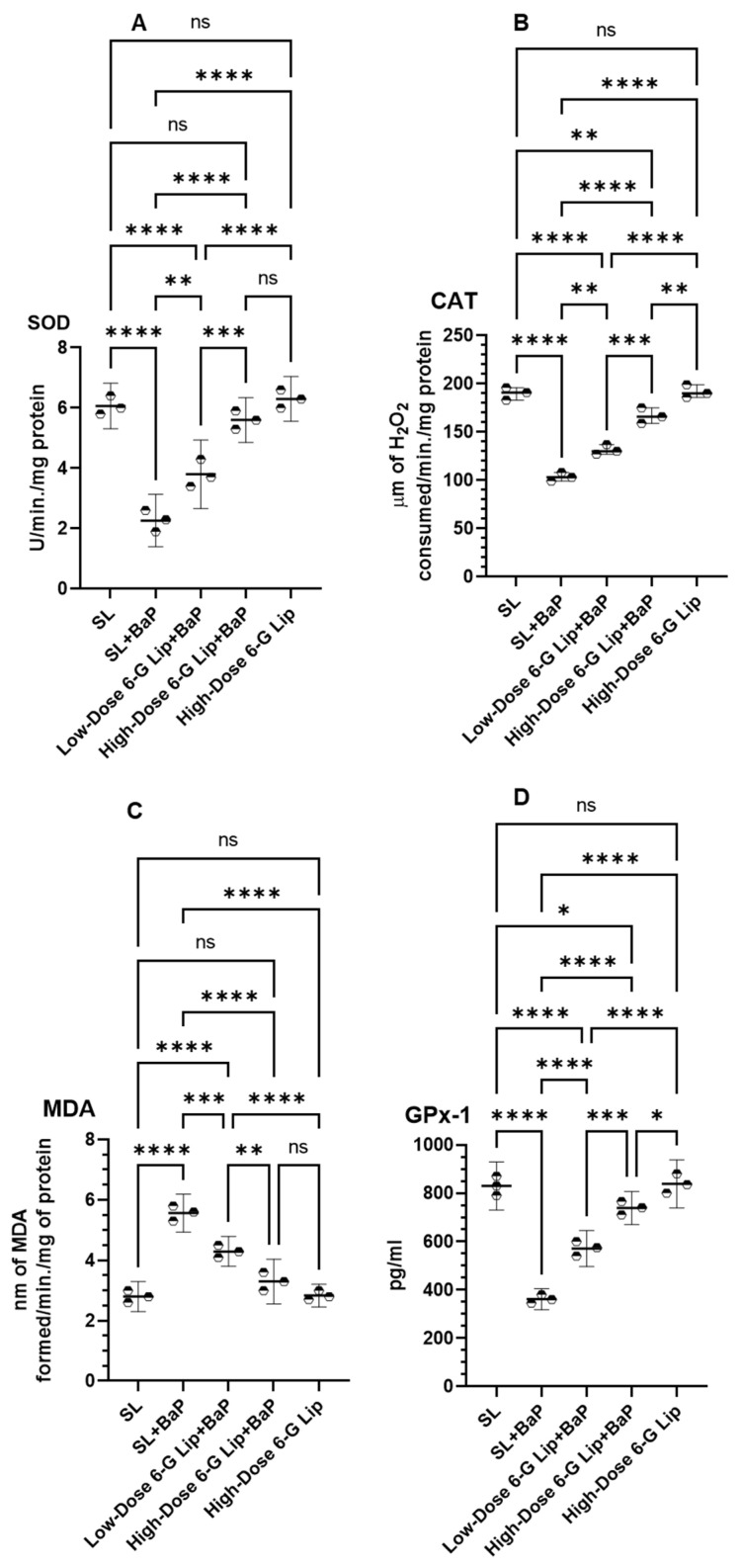
Protective effects of 6-G-Lip on antioxidant enzyme activities in BaP-induced lung carcinogenesis. This figure illustrates the impact of 6-G-Lip on oxidative stress regulation in lung tissues of BaP-induced small cell lung carcinoma (SCLC) mice. (**A**) Superoxide dismutase (SOD); (**B**) catalase (CAT); (**C**) malondialdehyde (MDA), a key marker of lipid peroxidation; and (**D**) glutathione peroxidase 1 (GPx1) across experimental groups. BaP exposure (SL + BaP) resulted in a marked depletion of SOD, CAT, and GPx1 activities, accompanied by a significant increase in MDA levels, reflecting severe oxidative stress and lipid peroxidation. Treatment with 6-G-Lip restored antioxidant enzyme activity in a dose-dependent manner, with high-dose 6-G-Lip + BaP achieving values comparable to the SL control group, indicating effective mitigation of BaP-induced oxidative damage. Notably, the high-dose 6-G-Lip-only group exhibited no significant deviations from the SL control, confirming its safety under physiological conditions. These findings underscore the potential of 6-G-Lip in counteracting oxidative stress-driven carcinogenesis. Data are presented as the mean ± 95% confidence intervals (CIs) from three independent experiments. Statistical significance is denoted as (*) *p* < 0.05, (**) *p* < 0.01, (***) *p* < 0.001, and (****) *p* < 0.0001; ‘ns’ indicates no statistically significant differences between groups.

BaP exposure led to a significant decline in antioxidant enzyme activity in the SL + BaP group, indicating oxidative stress-induced damage. The activity of superoxide dismutase (SOD) was reduced to 2.26 U/min/mg protein (95% CI: 1.39–3.13), catalase (CAT) activity dropped to 103.33 μm of H_2_O_2_ consumed/min/mg protein (95% CI: 92.13–114.53), and glutathione peroxidase-1 (GPx-1) levels decreased to 361.66 pg/mL (95% CI: 314.72–408.60). In contrast, the SL group exhibited significantly higher baseline levels of SOD (6.06 U/min/mg protein, 95% CI: 5.30–6.82), CAT (190.0 μm of H_2_O_2_ consumed/min/mg protein, 95% CI: 173.71–206.29), and GPx-1 (830.0 pg/mL, 95% CI: 730.63–929.36) (Figure 5A,B,D). Lipid peroxidation, measured by malondialdehyde (MDA) levels, was markedly increased in the SL + BaP group, reaching 5.56 nm of MDA formed/min/mg protein (95% CI: 4.83–6.29), compared to 2.80 nm of MDA formed/min/mg protein (95% CI: 2.30–3.29) in the SL group, reflecting severe oxidative damage (Figure 5C). Administration of [6]-G-Lip effectively restored antioxidant enzyme activity, counteracting BaP-induced oxidative stress. In the low-dose 6-G-Lip + BaP group, SOD activity increased to 3.80 U/min/mg protein (95% CI: 2.66–4.93), while in the high-dose 6-G-Lip + BaP group, it was further elevated to 5.60 U/min/mg protein (95% CI: 4.85–6.34). A similar effect was observed for CAT, which increased to 131.33 μm of H_2_O_2_ consumed/min/mg protein (95% CI: 118.58–144.08) and 166.66 μm of H_2_O_2_ consumed/min/mg protein (95% CI: 146.74–186.59) in the low-dose and high-dose 6-G-Lip + BaP groups, respectively. The levels of GPx-1 were also significantly elevated to 571.66 pg/mL (95% CI: 496.80–646.43) and 738.33 pg/mL (95% CI: 669.92–806.74), respectively. Furthermore, MDA levels, indicative of lipid peroxidation, were significantly reduced following [6]-G-Lip treatment. In the low-dose 6-G-Lip + BaP group, MDA levels declined to 4.3 nm of MDA formed/min/mg protein (95% CI: 3.80–4.79), whereas in the high-dose 6-G-Lip + BaP group, they further decreased to 3.3 nm of MDA formed/min/mg protein (95% CI: 2.55–4.04). The reduction in MDA and the restoration of antioxidant enzyme activity underscore the potent cytoprotective and antioxidative properties of [6]-G-Lip, reinforcing its chemopreventive potential in counteracting BaP-induced oxidative stress.

### 2.5. Reactive Oxygen Species (ROS) Analysis via Flow Cytometry

**Figure 6:**
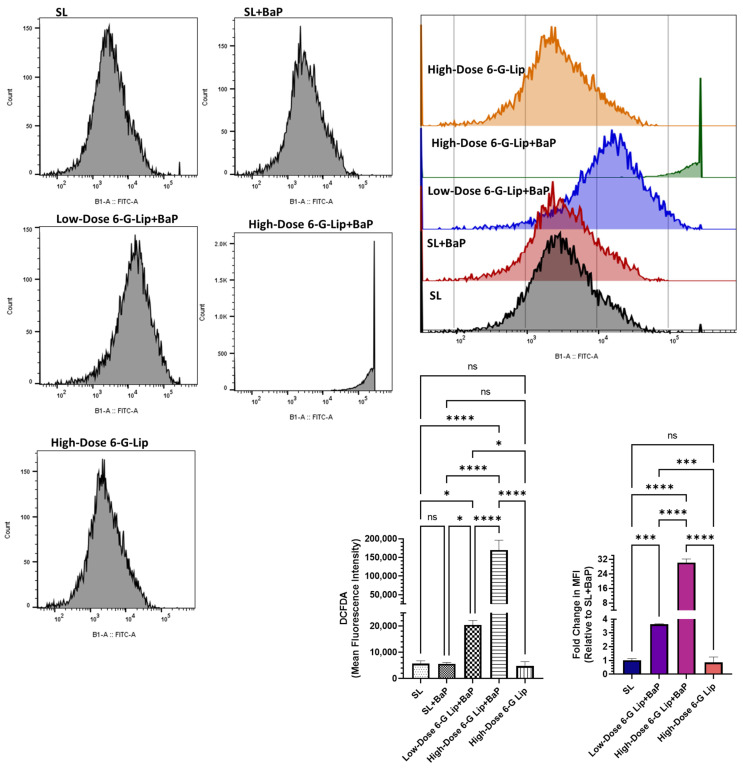
Effect of 6-G-Lip on intracellular reactive oxygen species (ROS) levels in lung cells. This figure illustrates the impact of 6-G-Lip on intracellular ROS levels in a BaP-induced SCLC model, as measured using the 2′,7′-dichlorofluorescein diacetate (DCFDA) assay via flow cytometry. The mean fluorescence intensity (MFI) represents ROS production across experimental groups. Exposure to BaP (SL + BaP) did not lead to a substantial increase in ROS compared to the vehicle control (SL), suggesting an adaptive cellular response that may facilitate tumor progression. In contrast, administration of 6-G-Lip resulted in a significant, dose-dependent elevation in ROS levels, with both low- and high-dose 6-G-Lip + BaP groups exhibiting markedly increased ROS accumulation relative to SL + BaP. This surge in ROS suggests that 6-G-Lip enhances oxidative stress in transformed cells, potentially driving apoptosis through ROS-mediated cytotoxicity. Notably, the high-dose 6-G-Lip-only group exhibited ROS levels comparable to those of the control, indicating that 6-G-Lip does not induce oxidative stress under non-malignant conditions. Data are presented as mean ± 95% confidence intervals (CIs) from three independent experiments. Statistical significance is denoted as (*) *p* < 0.05, (***) *p* < 0.001, (****) *p* < 0.0001, while ‘ns’ indicates no statistically significant difference between groups.

**Figure 7:**
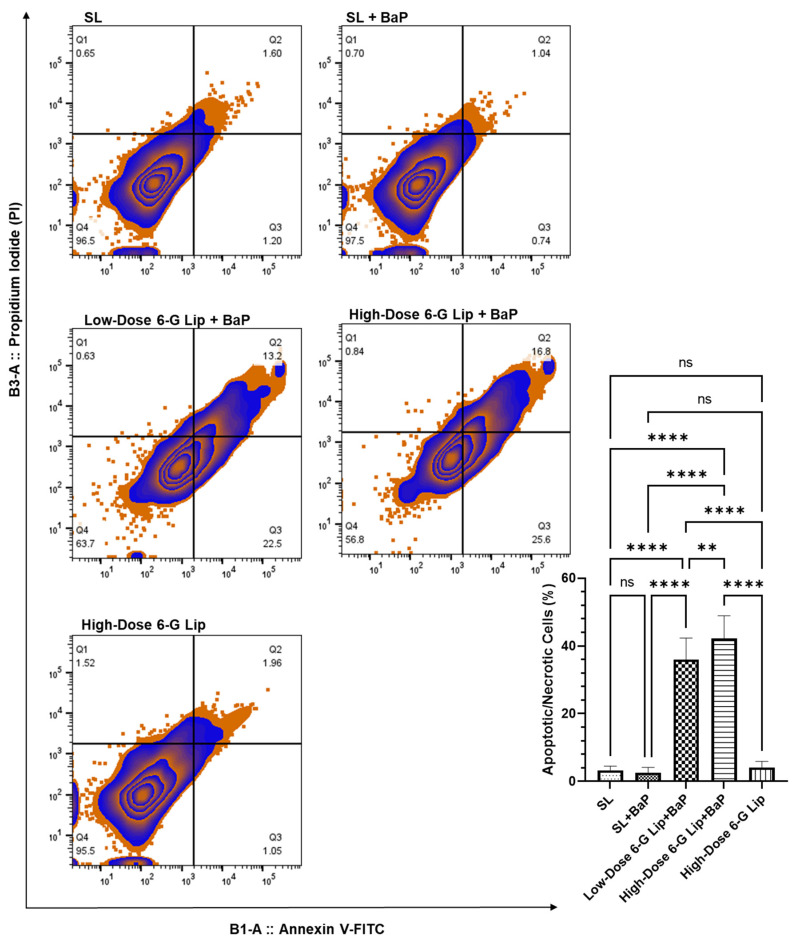
6-G-Lip-induced apoptosis in lung cells, evaluated by annexin V-FITC/PI flow cytometry. This figure illustrates the pro-apoptotic effects of 6-G-Lip in lung cells from a BaP-induced SCLC model, assessed using Annexin V-FITC and propidium iodide (PI) staining via flow cytometry. The analysis differentiates between viable, early apoptotic, late apoptotic, and necrotic cell populations across experimental groups. The SL + BaP group exhibited minimal apoptosis, indicating an inadequate activation of intrinsic cell death pathways in response to BaP-induced carcinogenesis. In contrast, 6-G-Lip treatment significantly increased apoptosis in a dose-dependent manner, with the low-dose 6-G-Lip + BaP group showing 36% apoptotic cells and the high-dose 6-G-Lip + BaP group reaching 42.2%, demonstrating a strong activation of programmed cell death mechanisms. The high-dose 6-G-Lip-only group exhibited apoptosis levels comparable to the vehicle control (SL), confirming that 6-G-Lip does not induce cytotoxicity under normal physiological conditions. Data are presented as mean ± 95% confidence intervals (CIs) from three independent experiments. Statistical significance is indicated as (**) *p* < 0.01, (****) *p* < 0.0001, while ‘ns’ denotes no statistically significant difference between groups.

The intracellular reactive oxygen species (ROS) levels in lung cells were quantified using DCFDA staining to evaluate oxidative stress. In the SL + BaP group, the mean fluorescence intensity (MFI) was 5626.66 (95% CI: 5116.76–6136.57), suggesting that oxidative stress remained relatively low despite BaP exposure, possibly due to adaptive cellular responses during SCLC progression (Figure 6). The ROS levels in this group were statistically comparable to those observed in the SL group (MFI: 5700; 95% CI: 4617.19–6782.90) and the high-dose 6-G-Lip-only group (MFI: 4766.67; 95% CI: 3075.75–6057.60), which did not receive BaP exposure. The absence of a significant difference between these groups indicates that high-dose 6-G-Lip alone did not induce oxidative stress in normal lung cells. Conversely, 6-G-Lip administration in the BaP-exposed groups resulted in a significant dose-dependent increase in ROS levels, suggesting its role in oxidative stress-mediated cytotoxicity. The low-dose 6-G-Lip + BaP group exhibited a marked elevation in ROS levels, with an MFI of 20,433.33 (95% CI: 18,779.31–22,087.35). A more pronounced increase was observed in the high-dose 6-G-Lip + BaP group, where MFI reached 169,666.66 (95% CI: 143,104.58–196,228.75). Compared to the SL + BaP group, the ROS levels in the low-dose 6-G-Lip + BaP and high-dose 6-G-Lip + BaP groups were elevated 3.63-fold (95% CI: 3.59–3.66) and 30.13-fold (95% CI: 28.11–32.14), respectively (Figure 7). These findings highlight the dual role of 6-G-Lip-induced oxidative stress in tumor suppression. While 6-G-Lip enhanced antioxidant defenses, as demonstrated in earlier analyses, it simultaneously promoted ROS accumulation in BaP-exposed groups, likely contributing to the induction of apoptosis in transformed cells. The biphasic role of ROS in cancer biology is well established, where moderate levels support cell survival, whereas excessive ROS accumulation triggers apoptosis. Flow cytometric analysis confirmed a significant increase in apoptosis in both the low-dose 6-G-Lip + BaP and high-dose 6-G-Lip + BaP groups (Figure 6). This dual regulatory effect highlights the therapeutic potential of 6-G-Lip, which effectively modulates oxidative homeostasis to suppress tumor progression in BaP-induced lung carcinogenesis.

### 2.6. Induction of Apoptosis in Lung Cells

The ability of 6-G-Lip to induce apoptosis was assessed using annexin V-FITC and PI staining, allowing for the differentiation of viable, early apoptotic, late apoptotic, and necrotic cells. Flow cytometric analysis revealed a substantial increase in apoptotic cell populations following 6-G-Lip treatment, confirming its role in programmed cell death induction (Figure 7). The high-dose 6-G-Lip + BaP group (G4) exhibited the highest apoptotic fraction, reaching 42.2% (95% CI: 35.52–48.88), indicating strong activation of apoptotic pathways. Similarly, the low-dose 6-G-Lip + BaP group showed a significant apoptotic response, with 35.96% (95% CI: 29.62–42.31), suggesting a dose-dependent increase in apoptosis. In contrast, the SL + BaP group demonstrated minimal apoptosis, indicating that BaP exposure alone could not trigger intrinsic cell death mechanisms, which may contribute to sustained carcinogenic progression. The notable apoptotic induction observed in 6-G-Lip-treated groups suggests that ROS accumulation is a key upstream signal, activating both mitochondrial (intrinsic) and death receptor-mediated (extrinsic) apoptotic pathways. The ability of 6-G-Lip to selectively induce apoptosis in pre-neoplastic and neoplastic cells while sparing normal lung tissue highlights its chemopreventive potential in counteracting BaP-induced SCLC. These findings underscore the dual role of 6-G-Lip as a modulator of oxidative stress and apoptosis, reinforcing its potential as a targeted therapeutic strategy to suppress lung carcinogenesis.

### 2.7. Histopathological Assessment of Lung Tissues

**Figure 8:**
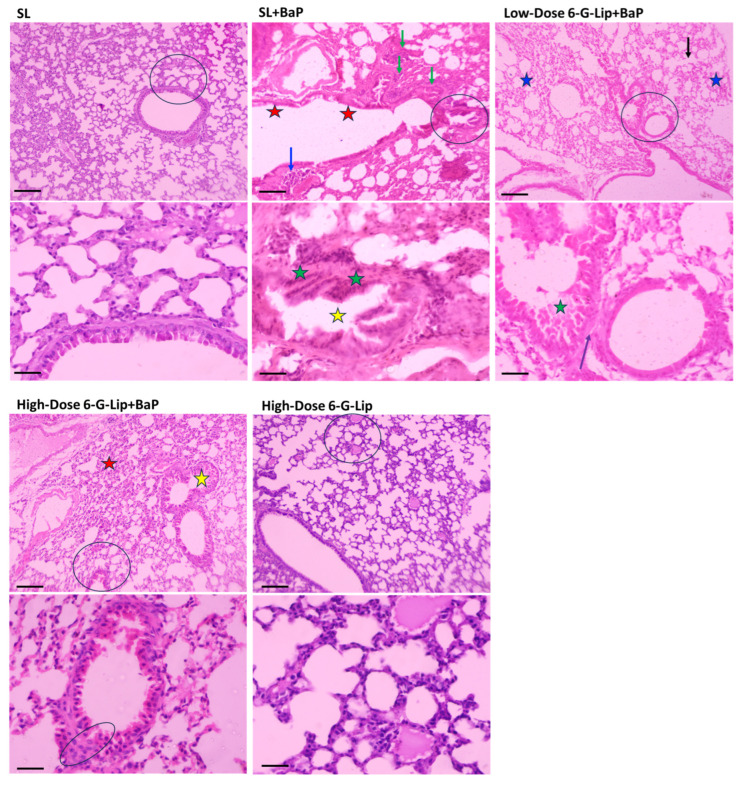
Histopathological assessment of lung tissues in BaP-induced SCLC and the effects of 6-G-Lip. Hematoxylin and eosin (H&E)-stained lung sections from experimental groups. SL + BaP group exhibits tumor cell proliferation, hemorrhagic regions (blue arrows), dilated bronchioles (red stars), and carcinoma invasion (green arrows). The lower panel highlights mitotic figures, hyperchromatic nuclei (green stars), and luminal narrowing (yellow stars). Low-dose 6-G-Lip + BaP group shows mild to moderate parenchymal alterations, hemorrhage (blue stars), emphysema (black arrow), and absence of carcinoma invasion (purple arrow). The high-dose 6-G-Lip + BaP group exhibits preserved lung architecture, characterized by mild chronic bronchitis (yellow star) and leukocytic infiltration with alveolar wall thickening (red star). SL and high-dose 6-G-Lip groups maintain normal bronchioles and alveolar structures. Upper panel: 100× magnification, bar = 100 µm; lower panel: 400× magnification, bar = 50 µm.

Histopathological evaluation revealed distinct pathological changes across experimental groups, providing critical insights into the chemopreventive potential of [6]-G-Lip against BaP-induced lung carcinogenesis (Figure 8). In the SL + BaP group, lung tissues exhibited extensive tumor formation, characterized by dense sheets of small, uniform tumor cells, a hallmark of small cell lung carcinoma (SCLC). Hemorrhagic regions (blue arrows) and dilated bronchioles (red stars) were prominent, reflecting significant structural disruption. Notably, carcinoma invasion (green arrows) was observed, further confirming the aggressive nature of BaP-induced malignancies. Magnified sections (lower panel) revealed intense tumor proliferation, mitotic figures, hyperchromatic nuclei (green stars), and luminal narrowing (yellow star), indicative of progressive neoplastic transformation. Conversely, the SL and high-dose 6-G-Lip groups exhibited preserved pulmonary architecture, with well-defined bronchioles, alveoli, and alveolar walls. No neoplastic lesions or structural abnormalities were detected, confirming that [6]-G-Lip alone does not induce adverse histopathological changes, reinforcing its safety in normal lung tissues. Lung sections from the low-dose 6-G-Lip + BaP group exhibited moderate parenchymal destruction, with necrotic regions and compression of adjacent alveoli; however, the overall tissue integrity remained largely intact. Fewer neoplastic lesions were observed in this group compared to the SL + BaP group, indicating partial protection. Mild hemorrhage (blue stars) and emphysematous changes (black arrow) were present, but carcinoma invasion was absent (purple arrow), suggesting that low-dose [6]-G-Lip attenuates tumor progression. The high-dose 6-G-Lip + BaP group demonstrated the most substantial protective effects, with minimal parenchymal destruction and markedly reduced tumor burden. Although chronic bronchitis (yellow star) and leukocytic infiltration with alveolar wall thickening (red star) were noted, these changes were significantly less severe than in the SL + BaP group. The lower panel confirmed only mild bronchiolar wall thickening, further emphasizing the ability of high-dose [6]-G-Lip to prevent the development of SCLC. Collectively, these findings provide strong histopathological evidence supporting the chemopreventive efficacy of [6]-G-Lip in mitigating BaP-induced pulmonary damage, highlighting its therapeutic potential for lung cancer prevention (Figure 8).

## 3. Discussions

This study comprehensively evaluates the chemopreventive potential of PEGylated [6]-Gingerol liposomes (6-G-Lip) in BaP-induced lung carcinogenesis. The findings demonstrate their ability to regulate oxidative stress, restore biochemical balance, induce apoptosis, and mitigate BaP-induced histopathological alterations. By leveraging advanced liposomal drug delivery technology, this study underscores the therapeutic promise of 6-G-Lip, supported by the well-documented anticancer properties of [6]-Gingerol.

The successful formulation and characterization of 6-G-Lip confirm its stability and efficacy as a nanocarrier system. The optimized formulation, with a particle size of 129.7 nm and a low polydispersity index of 0.16, ensures a uniform and monodisperse system, which is critical for biological applications. The zeta potential of −18.2 mV indicates strong colloidal stability, preventing aggregation and allowing for an extended circulation time. Notably, the high encapsulation efficiency of 91% highlights its exceptional drug-loading capacity, ensuring prolonged therapeutic availability. These attributes align with our previous studies on PEGylated liposomal formulations of thymoquinone (TQ), diallyl disulfide (DADS), and diallyl trisulfide (DATS), which demonstrated improved bioavailability, enhanced stability, and significant anticancer efficacy in preclinical models [34,36,37,38].

A critical aspect of the liposomal formulation is the stability and composition of the lipid bilayers, which dictate membrane rigidity, permeability, and drug retention [39,40,41,42]. In this study, we employed the DSPC-to-cholesterol molar ratio of 49:21, which we previously established to confer high stability and optimal drug retention in PEGylated liposomes [43]. This lipid ratio balances bilayer rigidity and fluidity, ensuring the liposomes remain structurally intact while facilitating controlled drug release. Cholesterol stabilizes liposomes by modulating membrane permeability and reducing premature drug leakage. The 2:1 phospholipid-to-cholesterol ratio is widely recognized as an effective composition for maximizing liposomal stability and integrity [44,45,46]. Our findings reaffirm that this optimized lipid composition stabilizes 6-G-Lip, contributing to its prolonged systemic circulation and sustained drug release profile.

A major challenge in nanocarrier-based drug delivery is the rapid clearance by the reticuloendothelial system (RES), which limits systemic circulation and drug availability at the tumor site. To overcome this, PEGylation was incorporated, allowing the liposomes to evade RES-mediated clearance, prolong systemic retention, and enhance tumor-specific accumulation. The controlled release profile of 6-G-Lip further underscores its therapeutic potential. Cumulative release studies revealed that only 26.5% of [6]-Gingerol was released in PBS and less than 50% in serum over 72 h at physiological temperature (37 °C) (Figure 2). This release pattern suggests that PEGylation prevents premature drug leakage, maintaining sustained drug levels at the target site. The slow and sustained release may be attributed to the lipophilic nature of [6]-gingerol, which interacts strongly with lipid bilayers, thereby reinforcing its retention within the liposomal core.

PEGylation also enhances tumor-specific drug accumulation through the enhanced permeability and retention (EPR) effect, which exploits tumors’ unique physiology to improve drug delivery efficiency [47,48]. The prolonged circulation and controlled release characteristics observed in this study align with well-established principles of nanocarrier-based drug delivery, reinforcing the pharmacokinetic advantages of PEGylated liposomes. By extending systemic retention and facilitating sustained drug release, 6-G-Lip can reduce and minimize systemic toxicity, thereby enhancing therapeutic efficacy.

A significant outcome of this study is the modulation of cancer marker enzymes, which serve as key indicators of BaP-induced carcinogenesis [49,50,51,52,53,54,55,56]. The substantial elevation in ADA, GGT, 5′-NT (CD73), and LDH following BaP exposure reflects tumor progression and metabolic alterations, which were effectively counteracted by 6-G-Lip administration. The ability of 6-G-Lip to restore normal enzyme levels suggests its potential to suppress cancer-associated metabolic dysregulation, consistent with previous studies demonstrating the anticancer potential of liposomal bioactive compounds [37,38].

The restoration of antioxidant defenses following the 6-G-Lip treatment represents another critical finding. BaP-induced oxidative stress significantly impaired SOD, CAT, and GPx-1 activity, leading to elevated MDA levels and lipid peroxidation. 6-G-Lip administration effectively restored these enzyme activities, mitigating oxidative stress-related damage. The increase in SOD activity from 2.26 U/min/mg protein (SL + BaP) to 5.60 U/min/mg protein (high-dose 6-G-Lip + BaP) reflects the antioxidative and cytoprotective effects of this formulation, similar to previous liposomal formulations of TQ and DATS, which also exhibited free radical scavenging and oxidative stress reduction [37,38].

A key mechanistic insight from this study is the dual role of 6-G-Lip in regulating oxidative stress. While 6-G-Lip restored antioxidant defenses in normal cells, it induced ROS accumulation in transformed cells, leading to the induction of apoptosis (Figure 7 and Figure 8). This selective regulation of oxidative stress is well-established in phytochemical-based anticancer strategies, where moderate ROS levels support tumor growth, but excessive accumulation triggers apoptosis [57,58,59,60,61]. The 3.63-fold and 30.13-fold increases in ROS levels in the low-dose and high-dose 6-G-Lip + BaP groups, respectively, confirm this biphasic response, suggesting that 6-G-Lip selectively promotes oxidative stress-mediated apoptosis in cancerous cells. Similar observations have been reported in our prior work on liposomal DATS and TQ formulations, which exhibited ROS-dependent apoptotic induction in colorectal and lung cancer models [37,38].

Apoptosis induction was a significant outcome of this study, further supporting the anticancer efficacy of 6-G-Lip. Flow cytometry analysis revealed a substantial increase in apoptotic cell populations, particularly in the high-dose 6-G-Lip + BaP group, where apoptosis reached 42.2%, compared to negligible levels in the SL + BaP group. This apoptotic response was driven by ROS accumulation, activating both mitochondrial (intrinsic) and death receptor-mediated (extrinsic) apoptotic pathways [59,61,62]. The absence of significant apoptosis in normal lung cells following 6-G-Lip treatment highlights its selective cytotoxicity, reinforcing its potential as a targeted anticancer intervention with minimal off-target effects. These findings align with our research on liposomal formulations of bioactive compounds, which demonstrated selective tumor cytotoxicity while preserving normal tissue integrity [37,38].

The histopathological assessment confirmed the protective effects of 6-G-Lip. BaP-induced lung tissue damage—characterized by alveolar disorganization, epithelial hyperplasia, and inflammatory infiltration was significantly reversed in the 6-G-Lip-treated groups, demonstrating restored alveolar architecture and decreased inflammation (Figure 8). These improvements emphasize the potential of 6-G-Lip in maintaining lung tissue integrity and inhibiting tumor progression, consistent with our previous findings using liposomal TQ-based formulations in lung cancer models [38]. In addition to localized pulmonary alterations, systemic manifestations such as body weight loss were also prominent in the BaP group (Figure 3A). While this can largely be attributed to tumor-induced metabolic stress, the extent of weight loss may also indicate metastatic spread, particularly to the liver. In our earlier BaP-induced models, we reported histopathological features that strongly suggest hepatic metastasis, including disrupted liver architecture, clusters of hyperchromatic cells with irregular nuclei and scant cytoplasm, and evidence of vascular invasion. Liver sections frequently exhibited sheets of malignant cells with high mitotic activity and infiltrative growth patterns [27,38]. Although hepatic tissues were not examined in this study, previous observations offer a plausible explanation for the cachexia-like phenotype observed here. This further emphasizes the systemic toxicity associated with BaP and highlights the protective potential of 6-G-Lip against both localized and systemic carcinogenic outcomes.

This study presents the first comprehensive investigation into the development, characterization, and in vivo evaluation of PEGylated liposomal [6]-gingerol (6-G-Lip) in a chemically induced lung cancer model. Although previous studies formulated [6]-gingerol using 1,2-dilauroyl-sn-glycero-3-phosphocholine (DLPC) and cholesterol for application in lung cancer systems, those efforts did not incorporate PEGylation, which is critical for enhancing systemic circulation and tumor-targeted delivery [63]. Furthermore, liposomes composed of hydrogenated soy phosphatidylcholine (HSPC) and cholesterol were evaluated in vitro against MDA-MB-231 breast cancer cells, without undergoing in vivo validation [64]. In contrast, the present study incorporates PEGylation and delivers a comprehensive preclinical evaluation, including detailed physicochemical characterization, selective modulation of ROS, induction of apoptosis, and reversal of BaP-induced histopathological alterations, collectively underscoring the potential of 6-G-Lip as a promising chemopreventive nanocarrier for lung cancer intervention.

The nanoformulation strategy employed in this study aligns with ongoing advancements in nanomedicine, particularly in the development of surface-engineered delivery systems designed to enhance therapeutic precision and bioavailability. These are comparable to recent innovations involving functionalized nanostructures, such as MnFe_2_O_4_ nanoparticles conjugated with myricetin to improve biological efficacy [65]. The PEGylated liposomal platform developed here provides a versatile foundation that could be adapted for other phytochemicals and disease models. This expands the translational relevance of the current formulation and opens promising avenues for future research in targeted chemoprevention.

## 4. Materials and Methods

### 4.1. Reagents

The primary reagents used in this study included distearoylphosphatidylcholine (DSPC) (Catalog # P1138), 1,2-distearoyl-sn-glycero-3-phosphatidylethanolamine-*N*-[methoxy(polyethylene glycol)-2000] (DSPE-PEG2000) (Catalog # 880120P), cholesterol (Chol) (Catalog # 228111), and [6]-gingerol ([6]-G) (Catalog # G1046), all obtained from Sigma-Aldrich (St. Louis, MO, USA). Additional reagents and assay kits were sourced from Abcam (Cambridge, MA, USA), including the Annexin V-FITC Apoptosis Detection Kit (ab14085), 2′,7′-dichlorofluorescin diacetate (DCFDA/H2DCFDA) Cellular ROS Assay Kit (ab113851), Lactate Dehydrogenase (LDH) Activity Assay Kit (ab102526), Gamma-Glutamyl Transferase (γ-GT) Assay Kit (ab241029), Superoxide Dismutase (SOD) Assay Kit (ab65354), Catalase (CAT) Assay Kit (ab83464), Lipid Peroxidation (MDA) Assay Kit (ab233471), and Glutathione Peroxidase 1 (GPx1) Assay Kit (ab10250). The carcinogenic agent benzo[a]pyrene (BaP) was purchased from Santa Cruz Biotechnology (Santa Cruz, CA, USA). Additionally, the 5′-Nucleotidase/CD73 Activity Assay Kit (Colorimetric) (Catalog # NBP3-24452) was obtained from Tocris Bioscience (Bio-Techne) in Bristol, UK.

### 4.2. Preparation of 6-Gingerol-Loaded Liposomes

PEGylated liposomes encapsulating [6]-gingerol (6-G-Lip) were prepared as described in our previous research [34,36,37,38]. This method involved mixing distearoylphosphatidylcholine (DSPC) and cholesterol (Chol) in a 7:3 molar ratio and adding 5% DSPE-PEG2000 to enhance liposomal stability and circulation time. The lipids were dissolved in chloroform, along with [6]-gingerol ([6]-G), to ensure effective encapsulation. The mixture was subjected to rotary evaporation under a nitrogen atmosphere to remove the organic solvent, resulting in a thin, uniform lipid film on the inner surface of the round-bottom flask. The dry lipid film was then hydrated with phosphate-buffered saline (PBS, pH 7.4) at room temperature, producing multilamellar vesicles (MLVs). The hydrated mixture was gently agitated to promote even dispersion of the lipid layers. To create unilamellar vesicles (ULVs) with a consistent size distribution, the MLV suspension was sonicated and then extruded through polycarbonate membranes with progressively smaller pore sizes, ranging from 400 nm to 100 nm.

### 4.3. Characterization of Liposomes

The physicochemical properties of the prepared PEGylated [6]-gingerol-loaded liposomes (6-G-Lip) were characterized by evaluating particle size, zeta potential, and polydispersity index (PDI) using dynamic light scattering (DLS) with the Zetasizer Nano system (Malvern Instruments, Malvern, Worcestershire, UK). This analysis provided critical insights into the liposomes’ size distribution and surface charge, which are essential parameters affecting their stability, biodistribution, and cellular uptake. The encapsulation efficiency (EE%) of [6]-gingerol was determined by lysing the liposomes with 0.5% Triton X-100, which released the encapsulated compound. The amount of [6]-gingerol released was quantified using a UV–vis spectrophotometer (Eppendorf, CT, USA) by measuring absorbance at 265 nm. The percentage of drug entrapment was calculated using the following equation:

$$\%\;\mathrm{Entrapment}\;\mathrm{Efficiency}\;\left(\mathrm{EE}\right)\;\mathrm{of}\;\mathrm{the}\;\mathrm{drug}=\frac{\mathrm{Liposome}\;\mathrm{entrapped}\;\mathrm{drug}}{\mathrm{Total}\;\mathrm{drug}}\times 100$$

### 4.4. In Vitro Stability and Release Studies

The in vitro stability of the PEGylated [6]-gingerol-loaded liposomes (6-G-Lip) was assessed by incubating the formulations in phosphate-buffered saline (PBS, pH 7.4) at 37 °C for 72 h. This incubation period was selected to evaluate the structural integrity and sustained release potential of the liposomal formulation under physiological conditions. To determine the release kinetics, 1 mL of the liposomal suspension was placed into dialysis bags with a molecular weight cutoff of 3.5 kDa and immersed in 20 mL of PBS supplemented with 9% sucrose. The setup was maintained at 37 °C with gentle agitation. At predetermined intervals, aliquots were withdrawn from the external release medium and replaced with an equal volume of fresh PBS to ensure constant volume and sink conditions. The concentration of released gingerol was quantified spectrophotometrically by measuring absorbance at 200 nm.

A similar procedure used 90% bovine serum to simulate biological fluid conditions and evaluate the release behavior in a more physiologically relevant environment. The cumulative drug release was calculated using the following equation:

$$\mathrm{Drug}\;\mathrm{release}\;(\%)=\frac{C_{n}V+\sum_{i=0}^{n}C_{i}V_{i}}{w}\times 100\%$$

In this equation, C*_n_* and C*_i_* represent the concentrations of [6]-G at the *n*-th and *i*-th time points, respectively. *V* is the total volume of the dialysis medium (20 mL), *V_i_* is the sampled volume taken at each time point (1 mL), and *w* denotes the total amount of [6] gingerol encapsulated within the liposomal formulation. This analysis enabled the evaluation of the release profile, offering essential insights into the liposomal system’s stability and sustained release capacity.

### 4.5. Animal Studies

#### 4.5.1. Ethical Considerations and Animal Handling

All animal experiments adhered to the ethical guidelines of the Animal Welfare Society at the University of London and were approved by the Qassim University Animal Ethics Committee (Approval No. 24-01-02, dated 19 August 2024). Female Swiss albino mice (8–10 weeks old) were housed under controlled conditions at King Saud University in Riyadh, Saudi Arabia, with a regulated 12 h light/dark cycle, standard laboratory chow, and water provided ad libitum. Animal welfare was prioritized throughout this study, with two daily health assessments conducted to monitor physical condition, activity, and signs of distress. Any signs of discomfort were promptly addressed in accordance with established ethical protocols.

#### 4.5.2. Experimental Design

**Figure 9:**
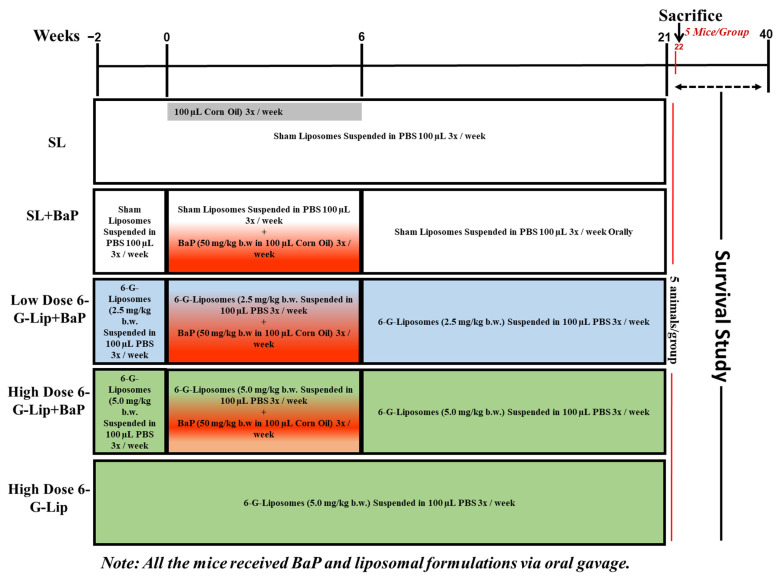
Experimental design overview. This figure illustrates the experimental design assessing the chemopreventive efficacy of [6]-gingerol-loaded PEGylated liposomes (6-G-Lip) in a benzo[a]pyrene (BaP)-induced small cell lung carcinoma (SCLC) model. Seventy-five female Swiss albino mice were randomly assigned to five groups (n = 15 per group) and received all treatments via oral gavage to ensure precise and consistent dosing. The vehicle control group (SL) received empty liposomes in phosphate-buffered saline (PBS) from week−2 to week 21. The SL + BaP group was administered BaP (50 mg/kg in corn oil) three times a week for four weeks to induce SCLC, and sham liposomes were administered. Two treatment groups received 6-G-Lip in a dose-dependent manner: low-dose 6-G-Lip + BaP (2.5 mg/kg) and high-dose 6-G-Lip + BaP (5.0 mg/kg), both administered from week 2 to week 21 alongside BaP. An additional high-dose 6-G-Lip group received 6-G-Lip (5.0 mg/kg) alone to evaluate its safety under normal physiological conditions. At week 22, five mice in each group were sacrificed for biochemical and histopathological assessments. The remaining ten mice in each group were monitored for survival analysis until week 40.

Seventy-five female Swiss albino mice were randomly assigned to five groups (n = 15 per group) to investigate the chemopreventive potential of [6]-gingerol-loaded PEGylated liposomes (6-G-Lip) against benzo[a]pyrene (BaP)-induced small cell lung carcinoma (SCLC), as illustrated in Figure 9. All treatments, including BaP, sham liposomes, and 6-G-Lip, were administered via oral gavage to ensure consistent and accurate dosing throughout this study. The vehicle control group (sham liposomes, SL) received empty liposomes suspended in 200 µL of phosphate-buffered saline (PBS), administered orally from week −2 to week 21. The BaP-treated group (SL + BaP) was administered BaP at a dose of 50 mg/kg body weight, dissolved in 200 µL of corn oil, three times a week for four weeks to induce SCLC, as per an established protocol [27,38]. This group also received sham liposomes, given in the same manner as those provided to the vehicle control group. Two treatment groups were established to assess the dose-dependent effects of 6-G-Lip. The Low-Dose 6-G-Lip + BaP group received 2.5 mg/kg of 6-G-Lip orally from week 2 to week 21, in conjunction with BaP administration, following the same schedule as the SL + BaP group. The high-dose 6-G-Lip + BaP group received 5.0 mg/kg of 6-G-Lip, administered concurrently with BaP exposure, following the same treatment timeline. An additional high-dose 6-G-Lip group was administered 5.0 mg/kg of 6-G-Lip from week 2 to week 21 to assess the formulation’s safety profile under normal physiological conditions without BaP induction. Five mice from each group were sacrificed at week 22 post-BaP exposure for biochemical and histopathological analyses to evaluate the chemopreventive potential of the treatments.

#### 4.5.3. Study of Average Body Weight (ABW) and Survival Rate

Throughout the experimental period, up to week 22, the animals’ average body weight (ABW) was recorded at baseline and monitored biweekly to track changes in body weight and assess general health status during treatment. Following this period, the remaining mice were monitored for survival outcomes, with observations continuing until week 40 post-BaP exposure to evaluate survival rates.

#### 4.5.4. Biochemical and Histological Analyses

##### Biochemical Marker Evaluation

Serum levels of cancer-associated biomarkers, including adenosine deaminase (ADA), lactate dehydrogenase (LDH), gamma-glutamyl transferase (GGT), and 5′-nucleotidase (CD73), were quantified using commercially available assay kits, following the manufacturers’ protocols. These biomarkers were analyzed to evaluate metabolic alterations and potential tissue damage associated with cancer progression.

##### Antioxidant Enzyme Activity

Lung tissue homogenates were prepared to evaluate the activity of key antioxidant enzymes, including superoxide dismutase (SOD), catalase (CAT), and glutathione peroxidase (GPx1). Lipid peroxidation was assessed by measuring malondialdehyde (MDA) levels, a recognized marker of oxidative stress and lipid membrane damage. Elevated MDA levels indicate the extent of oxidative injury caused by reactive oxygen species (ROS), providing valuable insights into the severity of tissue damage. For the analysis, approximately 25 mg of lung tissue was weighed and homogenized in 100 µL of the specific buffer provided with each assay kit. The homogenates were centrifuged at 10,000× *g* for 15 min at 4 °C, and the resulting supernatants were collected for biochemical analysis.

##### Histopathological Assessment of Lung Tissues

The histopathological evaluation of lung tissues investigated the chemopreventive potential of [6]-gingerol-loaded liposomes (6-G-Lip) against benzo[a]pyrene (BaP)-induced small cell lung cancer (SCLC). Immediately after collection, the lung tissues were fixed in 10% neutral-buffered formalin to preserve cellular morphology and structural integrity. The fixed samples underwent a standard dehydration process using a graded series of ethanol, were cleared in xylene, and embedded in paraffin wax to ensure proper tissue preservation for microscopic examination. Sections measuring 4–5 µm in thickness were sliced using a microtome (RM2235) (Leica Biosystems, Deer Park, IL, USA) to obtain uniform slices suitable for detailed histological analysis. These sections were stained with hematoxylin and eosin (H&E) to enhance cellular architecture and facilitate the visualization of pathological features. The stained slides were examined under a light microscope (Olympus CX43) at 100× magnification to identify histopathological changes indicative of tumor development and progression. The evaluation focused on identifying key pathological alterations, including hyperplasia, dysplasia, and other structural abnormalities characteristic of malignant transformation in lung tissue. This analysis provided critical insights into the protective effects of 6-G-Lip treatment against BaP-induced histological damage.

#### 4.5.5. Apoptosis and ROS Assays

##### Apoptosis Analysis

Flow cytometry was conducted to assess cell viability and identify the various stages of apoptosis: viable, necrotic, early apoptotic, and late apoptotic cells. For each experimental group, three randomly selected lung tissues were used. The tissues were processed into single-cell suspensions using the gentleMACS Dissociator (Miltenyi Biotec, Bergisch Gladbach, Germany). The resulting suspensions were filtered through a 100 µm mesh to remove debris and centrifuged at 300× *g* for 5 min. The cell pellet was washed twice with cold phosphate-buffered saline (PBS) and resuspended in Annexin V binding buffer at a final density of 1 × 10^6^ cells/mL. Sequentially, the staining was performed by incubating the cells with Annexin V-FITC and propidium iodide (PI) for 15 min at room temperature in the dark. The stained cells were then analyzed using the MACSQuant Analyzer 10 (Miltenyi Biotec, Bergisch Gladbach, Germany). Data were acquired using standard compensation and gating strategies to accurately differentiate cell populations. The results were analyzed using FlowJo software (version 10.9.0), which enabled the precise identification of apoptotic stages and the quantification of cell viability across the experimental groups.

##### Measurement of Intracellular ROS Production

Intracellular reactive oxygen species (ROS) levels were assessed using the fluorescent probe 2′,7′-dichlorofluorescein diacetate (DCFDA). Lung tissues were prepared as described previously, ensuring the preparation of consistent and uniform suspensions. The cells were then filtered through a 100 µm cell strainer, washed twice with PBS, and adjusted to a concentration of 1 × 10^6^ cells/mL in Dulbecco’s Modified Eagle Medium (DMEM) supplemented with 1% fetal bovine serum (FBS). The cells were incubated with 20 µM DCFDA for 40 min at 37 °C in a humidified incubator with 5% CO_2_. After incubation, the cells were washed with PBS to remove unbound dye and were analyzed immediately using the MACSQuant Analyzer 10. The mean fluorescence intensity (MFI) values were recorded for each sample, reflecting the intracellular levels of ROS. Data analysis was performed using FlowJo software (v10.9.0), generating histograms and comparing ROS production across different treatment groups. This analysis provided a quantitative assessment of oxidative stress levels and highlighted the potential protective effects of the tested formulations.

#### 4.5.6. Statistical Analysis

All data are presented as means with corresponding 95% confidence intervals (CIs). Comparisons between experimental groups were conducted using one-way or two-way analysis of variance (ANOVA), followed by Tukey’s post hoc test for multiple comparisons. Statistical analyses were performed using GraphPad Prism version 9. A *p*-value of <0.05 was deemed statistically significant.

## 5. Conclusions

This study presents the first successful development, characterization, and in vivo evaluation of PEGylated [6]-gingerol liposomes (6-G-Lip) in a BaP-induced lung carcinogenesis model. The findings demonstrate that 6-G-Lip effectively attenuates BaP-induced oxidative stress, restores biochemical and antioxidant balance, selectively induces apoptosis in transformed cells, and preserves lung tissue integrity. The high encapsulation efficiency, sustained release profile, and extended systemic circulation support its potential as a chemopreventive nanocarrier for lung cancer. Notably, the formulation exhibited selective cytotoxicity toward malignant cells while sparing normal tissues, underscoring its promise for safer and more targeted therapeutic intervention.

Despite these encouraging results, certain limitations must be acknowledged. The present study did not investigate the precise molecular pathways underlying the observed therapeutic effects, which remain to be elucidated in future mechanistic studies. Furthermore, detailed pharmacokinetic and biodistribution analyses are necessary to evaluate systemic exposure, tissue-specific accumulation, and long-term safety. These investigations will be crucial in supporting the translational potential of the formulation.

Future work should also consider alternative surface modification strategies to enhance tumor-targeting efficiency and extend the applicability of this platform to other cancer models. In conclusion, 6-G-Lip represents a significant advancement in nanotechnology-based cancer prevention, offering a stable, selective, and effective strategy for managing lung cancer. With further optimization and clinical validation, this nanoformulation holds strong potential to contribute to next-generation cancer therapeutics.

## Data Availability

The original contributions presented in this study are included in the article. Further inquiries can be directed to the corresponding author.
